# From QTL mapping to genome editing: advances and integrated strategies for improving aluminum tolerance in crops

**DOI:** 10.3389/fpls.2026.1862097

**Published:** 2026-06-03

**Authors:** Ying Liu, Chunrui Chen, Huihui Zhu, Jianli Yang

**Affiliations:** 1 College of Resources and Environment, Yunnan Agricultural University, Kunming, China; 2 Key Laboratory of Vegetable Biology of Yunnan Province, College of Landscape and Horticulture, Yunnan Agricultural University, Kunming, China

**Keywords:** acid soils, ALMT/MATE transporters, aluminum tolerance, CRISPR/Cas, genomic selection, GWAS, high-throughput phenotyping, QTL mapping

## Abstract

Aluminum (Al) toxicity in acidic soils remains one of the most serious constraints on global crop production, limiting productivity across nearly one-third of the world’s potentially arable land. As pressure grows to cultivate marginal lands under climate change and food security challenges, improving Al tolerance has become an urgent priority in crop science and sustainable agriculture. This review provides a timely synthesis of recent advances in the genetic and molecular dissection of Al tolerance, highlighting the progression from classical biparental QTL mapping to genome-wide association studies and, more recently, CRISPR/Cas-based precision editing. Major breakthroughs, including the identification of key ALMT and MATE transporters, the expansion of STOP1-centered regulatory networks, and the discovery of the first Al receptor, have greatly deepened our understanding of plant adaptation to acid soils. We further examine how high-throughput phenotyping, marker-assisted selection, genomic selection, and gene pyramiding are accelerating the translation of genetic discoveries into breeding practice. Importantly, emerging genome-editing strategies now enable targeted and potentially transgene-free improvement of endogenous tolerance genes. By integrating molecular breeding with agronomic approaches such as liming and nutrient management, this review outlines a forward-looking framework for developing resilient crop varieties and achieving more sustainable productivity on acidic soils worldwide.

## Introduction

Aluminum (Al) is the most abundant metal in the Earth’s crust, and its phytotoxic trivalent form, Al^3+^, becomes soluble when soil pH falls below 5.0-5.5. Because 30-40% of the world’s potentially arable land is acidic, Al toxicity represents a major global limitation to crop production (von Uexküll and Mutert, 1995; Kochian et al., 2015; Ofoe et al., 2023; Rahman et al., 2018). One of its earliest and most characteristic symptoms is the rapid inhibition of root elongation, which severely constrains the uptake of water and nutrients, reduces biomass accumulation, and ultimately lowers yield (Ofoe et al., 2023; Kocjan et al., 2025). In cereals, severe Al toxicity alone may reduce yields by as much as 30-40% under extreme conditions, although actual losses vary widely with species, genotypes, and soil properties (Inostroza-Blancheteau et al., 2010; Too et al., 2020).

Aluminum tolerance is a quantitatively inherited trait governed by both major and minor genes, and its expression is strongly shaped by environmental conditions such as soil pH, Al concentration, and nutrient availability (Rahman et al., 2018; Zhu and Shen, 2024). Although liming is widely practiced to mitigate soil acidity, its effects are often confined to the topsoil, and the associated costs can be prohibitive, particularly in low-input agricultural systems. In addition, liming is generally ineffective against subsoil acidity (Kochian et al., 2004; Enesi et al., 2023). For these reasons, genetic improvement for Al tolerance has become an attractive and sustainable strategy for enhancing crop productivity on acidic soils (Delhaize and Ryan, 1995; Ma et al., 2001; Zhu and Shen, 2024).

### Classical tolerance mechanisms: organic acid exudation via ALMT and MATE transporters

**Figure 1:**
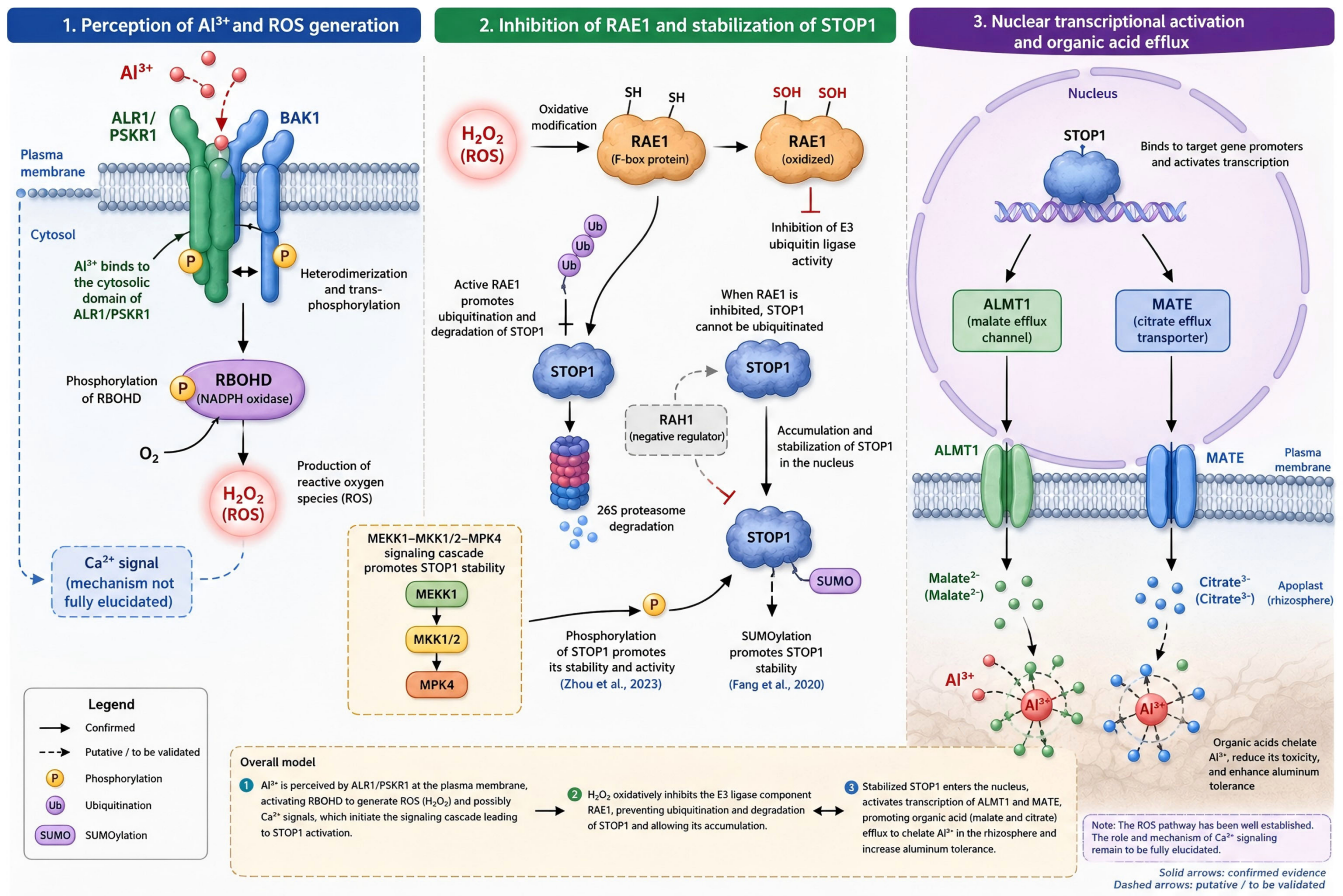
ALR1/PSKR1-mediated aluminum signal transduction and tolerance mechanism in plants. Aluminum ions (Al³^+^) are perceived by the ALR1/PSKR1 receptor complex at the plasma membrane, where ALR1/PSKR1 interacts with the co-receptor BAK1 and undergoes trans-phosphorylation. Activated ALR1/PSKR1 subsequently phosphorylates the NADPH oxidase RBOHD, leading to the production of reactive oxygen species (ROS), primarily H_2_O_2_. A potential Ca²^+^ signaling branch downstream of ALR1/PSKR1 is indicated with dashed arrows because the underlying mechanism remains incompletely understood. Elevated H_2_O_2_ oxidatively modifies the F-box E3 ubiquitin ligase component RAE1, thereby suppressing its ubiquitination activity toward the transcription factor STOP1. Inhibition of RAE1 prevents STOP1 degradation via the 26S proteasome, resulting in STOP1 accumulation and stabilization in the nucleus. The negative regulator RAH1 may additionally contribute to STOP1 repression. Furthermore, the MEKK1–MKK1/2–MPK4 kinase cascade phosphorylates STOP1 to enhance its stability, while SUMOylation further promotes STOP1 accumulation and activity. Stabilized STOP1 activates transcription of aluminum tolerance genes, including *ALMT1* and *MATE*, encoding malate and citrate efflux transporters, respectively. Secretion of malate and citrate into the rhizosphere chelates toxic Al³^+^ ions, thereby reducing aluminum toxicity and enhancing plant aluminum tolerance. Solid arrows indicate experimentally supported pathways, whereas dashed arrows denote putative or incompletely resolved mechanisms.

The most important Al tolerance genes identified so far belong to two transporter families: the aluminum-activated malate transporters (ALMTs) and the multidrug and toxic compound extrusion (MATE) proteins. These transporters facilitate the release of malate and citrate, respectively, from root apices (**Figure 1**). Once secreted, these organic acid anions chelate Al^3+^ in the rhizosphere and thereby prevent its entry into root cells (Ryan et al., 2001; Yang et al., 2019). Representative examples include TaALMT1 in wheat (Sasaki et al., 2004), SbMATE in sorghum (Magalhaes et al., 2007), ZmMATE1 in maize (Maron et al., 2010), OsFRDL4 in rice (Yokosho et al., 2011), and HvMATE in barley (Furukawa et al., 2007; Nguyen Thao et al., 2025). Each of these genes has been functionally characterized and is now recognized as a major determinant of Al tolerance in its respective crop.

### Transcriptional control: the STOP1-centered regulatory network

Over the past twenty years, substantial advances have been made in understanding how plants perceive and respond to Al stress. A complex regulatory network centered on the transcription factor STOP1 (Sensitive to Proton Rhizotoxicity 1) has been shown to control the expression of numerous downstream genes involved in tolerance (Huang and Ma, 2025). Beyond STOP1, additional regulatory layers are increasingly being recognized, including protein turnover, epigenetic regulation (e.g. DNA methylation and histone modifications at Al-responsive loci), and signaling pathways (Kocjan et al., 2025).

### From Al perception to transcriptional response: the ALR1 signaling pathway

Particularly notable is the recent identification of the first Al receptor ALR1 (Aluminum Resistance 1) (Ding et al., 2024; Ryan and Yang, 2024), which provides new insight into how plants sense toxic Al ions at the cell surface. The discovery of PSKR1, also known as ALR1, as an intracellular Al³^+^ sensor has substantially reshaped current understanding of Al perception. Loss-of-function mutations in *PSKR1* compromise STOP1 nuclear accumulation and *AtALMT1* expression (Ding et al., 2024). Al³^+^ binds directly to the cytoplasmic domain of PSKR1, promoting recruitment of the co-receptor BAK1 and inducing mutual phosphorylation that activates the receptor complex. Once activated, PSKR1 phosphorylates the plasma membrane NADPH oxidase RBOHD, thereby stimulating reactive oxygen species (ROS) production. These ROS oxidatively modify Cys364 of the F-box protein RAE1 and suppress its E3 ubiquitin ligase activity. Inhibition of RAE1 stabilizes the transcription factor STOP1, leading to increased expression of Al tolerance genes, including *AtALMT1*. In addition, the receptor-like cytoplasmic kinase PBL27 phosphorylates PSKR1 at Ser696/698 and negatively regulates PSKR1-mediated signaling (Xu et al., 2025). Together, these findings define a direct, phosphorylation-dependent signaling cascade that links intracellular Al sensing to STOP1 stabilization, rather than an extracellular or plasma membrane-initiated perception event. A working model integrating ALR1, ROS/Ca²^+^ signals, and the STOP1–ALMT/MATE axis is presented in **Figure 1**. Further clarification of the complete signaling relay from ALR1 to STOP1 remains an important objective for future research.

### Methodological advances in genetic dissection

Alongside these biological discoveries, methodological progress has transformed the study and improvement of Al tolerance. Early investigations relied on classical linkage analysis and quantitative trait locus (QTL) mapping in biparental populations, which identified major genomic regions associated with tolerance (Ryan et al., 2010; Bezie et al., 2025). The subsequent development of genome-wide association studies (GWAS) enabled higher-resolution analysis of natural allelic variation across diverse germplasm collections (Famoso et al., 2011; Cai et al., 2013; Jaiswal et al., 2024). More recently, CRISPR/Cas-based genome editing has opened the possibility of modifying endogenous genes with high precision, allowing targeted enhancement of Al tolerance without necessarily introducing foreign DNA (Bhattacharjee et al., 2023; Cai et al., 2025).

### Critical note on translational limitations

While *Arabidopsis thaliana* has provided invaluable mechanistic insights (e.g., *AtALMT1*, *STOP1*, *RAE1/RAH1*), direct translation to crops requires caution. Differences in root architecture, the relative contribution of apoplastic versus symplastic Al uptake, and the dominance of exclusion versus internal tolerance mechanisms vary substantially between *Arabidopsis* and cereals such as rice or maize. Therefore, findings from the model species should be validated in the target crop before breeding applications.

### Scope and objectives of this review

**Figure 2:**
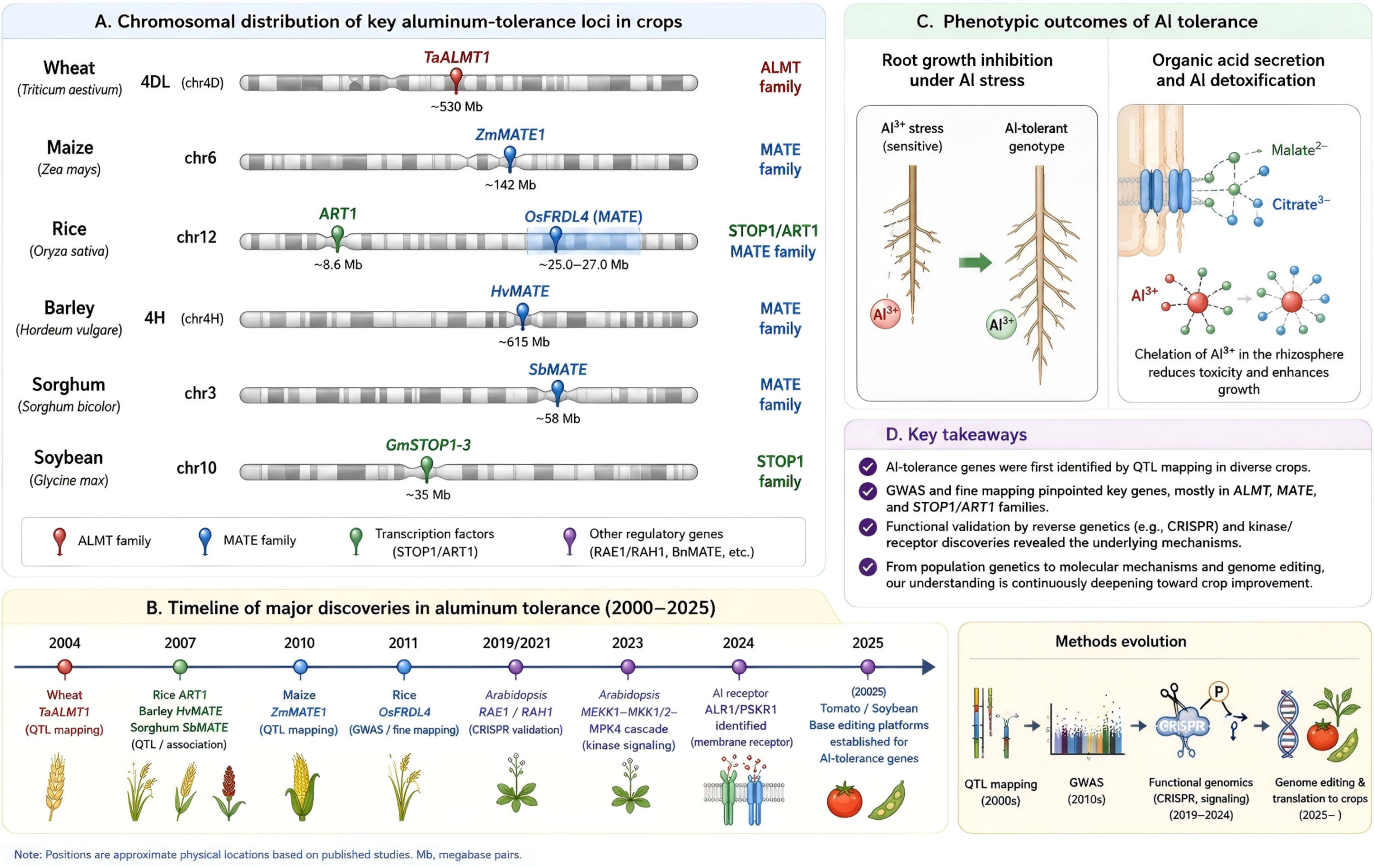
Major aluminum-tolerance QTLs and GWAS discoveries: a spatiotemporal panorama. **(A)** Chromosomal distribution of representative aluminum-tolerance loci in crops. Representative chromosomes from wheat (*Triticum aestivum*), maize (*Zea mays*), rice (*Oryza sativa*), barley (*Hordeum vulgare*), sorghum (*Sorghum bicolor*), and soybean (*Glycine max*) are shown with approximate physical positions of major Al-tolerance genes and QTLs. *TaALMT1* located on wheat chromosome 4DL represents the ALMT transporter family. Members of the MATE transporter family include *ZmMATE1* (maize chromosome 6), *OsFRDL4* (rice chromosome 12), *HvMATE* (barley chromosome 4H), and *SbMATE* (sorghum chromosome 3). Transcriptional regulators include *ART1* in rice and *GmSTOP1–3* in soybean. Different colors denote distinct functional classes: red, ALMT family; blue, MATE family; green, transcription factors (STOP1/ART1 family); purple, other regulatory components such as *RAE1*, *RAH1*, and *BnMATE*. Chromosomal positions are approximate and based on published genome assemblies. **(B)** Timeline of major discoveries in aluminum tolerance research (2000–2025). The timeline illustrates the progression of Al-tolerance research from classical QTL mapping to functional genomics and genome editing. Key milestones include identification of *TaALMT1* in wheat (2004), discovery of *ART1*, *HvMATE*, and *SbMATE* (2007), characterization of *ZmMATE1* in maize (2010), fine mapping of *OsFRDL4* in rice (2011), functional validation of *RAE1* and *RAH1* through reverse genetics and CRISPR approaches (2019/2021), identification of the MEKK1–MKK1/2–MPK4 signaling cascade regulating STOP1 stability (2023), discovery of the ALR1/PSKR1 aluminum receptor system (2024), and the establishment of conceptual base-editing platforms for aluminum-tolerance improvement in tomato and soybean (2025). **(C)** Phenotypic consequences of aluminum tolerance mechanisms. Aluminum-sensitive roots exhibit strong inhibition of root elongation under Al stress, whereas tolerant genotypes maintain root growth through activation of detoxification mechanisms. Organic acids such as malate and citrate are secreted into the rhizosphere through ALMT and MATE transporters, where they chelate toxic Al³^+^ ions and reduce aluminum toxicity. **(D)** Conceptual progression of research methodologies. Advances in aluminum-tolerance studies have evolved from QTL mapping and linkage analysis to GWAS, functional genomics, CRISPR-based validation, receptor/signaling studies, and genome editing–assisted crop improvement. Together, these developments have progressively deepened our understanding of the genetic and molecular basis of plant aluminum tolerance.

Given the rapid proliferation of studies on Al tolerance genetics, this review aims to provide a systematic and critical synthesis of recent advances, with a particular focus on the progression from classical QTL mapping to GWAS and, more recently, CRISPR/Cas-based precision editing. Specifically, we: (i) summarize key discoveries from biparental QTL mapping that established the foundational genetic framework for Al tolerance across major crops; (ii) evaluate how GWAS has captured natural allelic variation and enhanced mapping resolution; (iii) discuss emerging genome-editing strategies, clearing distinguishing demonstrated applications from promising but not yet validated approaches, that enable precise, potentially transgene-free improvement of endogenous tolerance genes; (iv) highlight the role of high-throughput phenotyping in accelerating genetic dissection; and (v) outline the integration of molecular breeding (MAS, GS, gene pyramiding) with agronomic management for sustainable productivity on acidic soils. By comparing the strengths and limitations of each approach, this review provides a forward-looking framework for researchers and breeders aiming to develop Al-resilient crop varieties. A comprehensive list of known Al tolerance genes is provided in **Table 1**, and the major QTL/GWAS discoveries are summarized schematically in **Figure 2**.

**Table 1:** Major genes, transporters, and regulators associated with aluminum tolerance in crops.

Gene	Crop species	Protein family/function	Mechanism	Identification method	Selected reference(s)
*TaALMT1*	Wheat (*Triticum aestivum*)	ALMT family, malate transporter	Al exclusion (malate efflux)	QTL (4DL), positional cloning	Sasaki et al., 2004; Ryan et al., 2010
*TaMATE1B*	Wheat	MATE family, citrate transporter	Al exclusion (citrate efflux)	QTL, association mapping	Tovkach et al., 2013; Pereira et al., 2015
*ZmMATE1*	Maize (*Zea mays*)	MATE family, citrate transporter	Al exclusion	QTL (bin 6.00), GWAS (CNV)	Maron et al., 2010, 2013
*ZmMATE2*	Maize	MATE family, unknown function	Not Al-induced (different mechanism)	QTL (bin 5.02-5.03)	Maron et al., 2010
*OsFRDL4*	Rice (*Oryza sativa*)	MATE family, citrate transporter	Al exclusion	QTL, functional expression	Yokosho et al., 2011, 2016
*OsART1*	Rice	C2H2-type zinc finger transcription factor	Transcriptional regulation of multiple genes	QTL, mutant analysis	Yamaji et al., 2009
*OsSTAR1/STAR2*	Rice	ABC transporter complex (nucleotide sugar transport)	Cell wall modification (UDP-glucose)	GWAS, mutant analysis	Huang et al., 2009; Famoso et al., 2011
*OsNrat1*	Rice	NRAMP family, Al³^+^ transporter	Uptake and internal detoxification	GWAS (aus subgroup)	Xia et al., 2010; Famoso et al., 2011
*OsALS1*	Rice	Half-size ABC transporter (tonoplast)	Internal sequestration (vacuolar)	Functional complementation	Huang et al., 2012
*HvMATE (HvAACT1)*	Barley (*Hordeum vulgare*)	MATE family, citrate transporter	Al exclusion	QTL (4H), positional cloning	Furukawa et al., 2007; Wang et al., 2007; Ma et al., 2016
*SbMATE*	Sorghum (*Sorghum bicolor*)	MATE family, citrate transporter	Al exclusion	QTL (AltSB, chr.3), positional cloning	Magalhaes et al., 2007
*GmSTOP1-3*	Soybean (*Glycine max*)	C2H2 zinc finger transcription factor	Flavonoid biosynthesis, ROS reduction	GWAS, transgenic validation	Liu et al., 2024
*GmPrx145*	Soybean	Class III peroxidase	ROS scavenging, cell wall modification	Fine mapping (qAl-HC2)	Cai et al., 2019
*BnALMT1/MATE*	Rapeseed (*Brassica napus*)	ALMT/MATE families	Al exclusion (organic acid efflux)	GWAS, QTL, transcriptomics	Gao et al., 2021; Zhou et al., 2022; Du et al., 2022
*PvALMT/MATE*	Common bean (*Phaseolus vulgaris*)	ALMT/MATE families	Putative exclusion	GWAS, QTL	Ambachew and Blair, 2021; Njobvu et al., 2020
*CaALMT2/4*	Chickpea (*Cicer arietinum*)	ALMT/MATE families	Putative exclusion	GWAS (first report)	Jia et al., 2025
*MtALMT8/9*	Medicago (*Medicago truncatula*)	ALMT family	Malate efflux	Genome-wide analysis, complementation	Jin et al., 2024
*AtALMT1*	*Arabidopsis thaliana*	ALMT family, malate transporter	Al exclusion	QTL, positional cloning	Hoekenga et al., 2006
*AtMATE*	*Arabidopsis thaliana*	MATE family	Al exclusion (citrate)	GWAS (local population)	Nakano et al., 2020b
*STOP1*	*Arabidopsis* (and orthologs in crops)	C2H2 zinc finger transcription factor	Master regulator of Al tolerance genes	Mapping, functional analysis	Iuchi et al., 2007; Huang and Ma, 2025
*RAE1/RAH1*	*Arabidopsis*	F-box proteins (ubiquitin E3 ligase components)	Regulate STOP1 stability (trade-off control)	Forward genetics, CRISPR	Zhang et al., 2019b; Fang et al., 2021
*VuMATE1*	Rice bean (*Vigna umbellata*)	MATE family, citrate transporter	Al-inducible, root-tip specific expression	Functional characterization	Liu et al., 2013, 2016

## Classical linkage analysis and QTL mapping: laying the foundation

Before the advent of high-throughput genotyping, classical linkage analysis using biparental segregating populations such as F_2_, recombinant inbred lines (RILs), and doubled haploids (DH) was the principal strategy for dissecting the genetics of Al tolerance. Although these approaches were constrained by limited allelic diversity and relatively low mapping resolution, they established the first genetic frameworks for Al tolerance and provided the basis for later gene cloning and marker development (Delhaize and Ryan, 1995; Kochian et al., 2004; Li et al., 2025b).

The genetic architecture of Al tolerance differs notably between monocots and dicots. In many monocot species, major QTLs correspond to genes encoding organic acid transporters, whereas dicots more often display polygenic inheritance involving multiple minor-effect loci and epistatic interactions. Below, key QTL mapping studies are reviewed, beginning with monocots and then turning to dicots.

### Milestone QTL mapping studies in major crops

Wheat has been one of the best-studied systems for Al tolerance. Early linkage analyses identified major loci on chromosome 4DL, including *Alt2* in ‘Chinese Spring’ and *AltBH* in ‘BH 1146’ (Milla and Gustafson, 2001), which were later associated with *TaALMT1* (Sasaki et al., 2004; Ryan et al., 2010). Additional studies in diverse genetic backgrounds revealed a combination of major and minor QTLs (Zhou et al., 2007). Synthetic hexaploid wheat has also proven valuable for uncovering novel loci derived from wild progenitors (Emebiri et al., 2020).

In maize, Al tolerance is genetically complex. Piñeros et al. (2005) compared six genotypes with contrasting Al tolerance and found no consistent relationship between root tip citrate exudation and Al resistance. Subsequent positional cloning identified two MATE members: *ZmMATE1* on chromosome 6 (bin 6.00), which co-segregated with a major Al tolerance QTL, and *ZmMATE2* on chromosome 5 (bin 5.02-5.03), corresponding to another major locus (Maron et al., 2010). *ZmMATE1* is strongly induced by Al in root tips, encodes a plasma membrane citrate transporter, and enhances Al tolerance when expressed in Arabidopsis. By contrast, *ZmMATE2* is not induced by Al and does not appear to function in citrate transport, implying a different mechanism. A later study using a tropical F_2:3_ population identified nine Al-tolerance QTLs distributed across chromosomes 2, 4, 5, 6, 7, 8, 9, and 10, together accounting for 70.3% of the phenotypic variation. Three major QTLs on chromosomes 6, 8, and 10 explained 40.3% of this variation, reinforcing the importance of the chromosome 6 region containing *ZmMATE1* while also confirming the polygenic nature of Al tolerance in maize (Coelho et al., 2019).

In rice, QTL mapping with recombinant inbred lines from ‘Asominori’ (japonica) × ‘IR24’ (indica) identified three major loci, *qRRE-1*, *qRRE-9*, and *qRRE-11*, each explaining 13.5-17.7% of the variation in relative root elongation under Al stress (Xue et al., 2006), with their effects confirmed by chromosome segment substitution lines. Subsequent GWAS revealed pronounced subpopulation differences, with japonica varieties exhibiting approximately twice the Al tolerance of indica, and uncovered additional loci that co-localized with known Al-sensitive mutants including *ART1*, *STAR2*, and *Nrat1* (Famoso et al., 2011). A recent meta-analysis integrating 12 mapping studies and five GWAS datasets refined 28 meta-QTLs, identified 219 differentially expressed candidate genes, and highlighted the role of transporters and transcription factors in Al tolerance (Jaiswal et al., 2024). Together, these studies support a polygenic model and provide strong candidates for marker-assisted breeding.

Barley QTL mapping has consistently revealed a major Al tolerance locus on chromosome 4H. Furukawa et al. (2007) and Wang et al. (2007) independently fine-mapped Alp to this chromosome and identified HvAACT1 (HvMATE), a citrate transporter belonging to the MATE family. Using a DH population from CXHKSL (moderately tolerant) × Gairdner (sensitive), Ma et al. (2016) detected a major 4H QTL that accounted for 71% of the observed variation and identified a new HvAACT1 allele characterized by a polymorphic 5′ UTR. In a different DH population, however, Navakode et al. (2009) reported additional QTLs on chromosomes 2H, 3H, and 4H, indicating that smaller-effect polygenic components may also contribute, depending on the genetic background.

In sorghum, a major Al tolerance locus, AltSB, was mapped to chromosome 3 (Magalhaes et al., 2004). Positional cloning later identified SbMATE, an Al-activated citrate transporter in the MATE family, as the underlying gene (Magalhaes et al., 2007). Regulatory polymorphisms, including a MITE insertion, were shown to enhance SbMATE expression in root apices of tolerant genotypes. Although AltSB is not orthologous to the group-4 Al tolerance genes of Triticeae species such as wheat, barley, and rye, it converges functionally on citrate-mediated Al exclusion. Field trials demonstrated clear agronomic value: the favorable allele increased grain yield by 0.6 t ha-1 on acid soils without penalty under non-toxic conditions, and even a single copy conferred a 0.5 t ha-1 yield gain in hybrids (Carvalho et al., 2015).

Soybean displays a more complex genetic architecture than many cereals. Early studies detected multiple QTLs of modest effect across the genome (Cai et al., 2019; Xiang et al., 2022). Subsequent analyses showed that both additive and epistatic effects contribute substantially to Al tolerance (Korir et al., 2011), and segregation analysis suggested the presence of two major genes in addition to polygenes (Qi et al., 2008). Fine mapping reduced key QTL intervals and facilitated the identification of candidate genes, including a citrate synthase homolog (Abdel-Haleem et al., 2014) as well as genes encoding glutathione S-transferase and peroxidase (Cai et al., 2019).

In rapeseed (*Brassica napus*), classical QTL mapping has been less extensive than in cereals, but several important studies have nonetheless been reported. In an RIL population derived from ‘10D130’ (tolerant) × ‘Zhongshuang 11’ (sensitive), Wang et al. (2020) identified 23 QTLs for germination-related traits distributed across the A and C genomes, each explaining 7.7-13.1% of the variation. Candidate gene mining highlighted 30 genes, including ALMT1, MATE, and transcription factors such as STOP1, NAC, and RAP2.4. Li et al. (2023) combined QTL mapping with transcriptomic and metabolomic analyses in 138 RILs, identifying 3,186 quantitative trait genes and 138 hub genes associated with 30 metabolites involved in lipid, carbohydrate, and secondary metabolism. Li et al. (2025a) integrated weighted gene co-expression network analysis (WGCNA) with QTL mapping and identified 26 key genes across 11 QTL regions, including qRDW-A09-1, qRDW-A10-1, and qRGV-A01-2, which were implicated in transcriptional regulation, stress responses, redox homeostasis, hormone signaling, cell wall modification, and calcium signaling. Using BSA-seq in an F2 population from ‘FDH188’ (tolerant) × ‘FDH152’ (sensitive), Zhou et al. (2024) detected three QTLs associated with taproot elongation and screened 55 candidate genes, including those encoding an ABC transporter G, zinc finger proteins, NAC, and ERF. Collectively, these findings indicate that Al tolerance in rapeseed is polygenic and distributed across both subgenomes, and they illustrate the value of multi-omics integration for dissecting complex traits.

In common bean (*Phaseolus vulgaris*), classical QTL mapping identified multiple loci involved in root morphology and Al resistance. López-Marín et al. (2009) detected 24 QTLs for root architecture traits under Al stress in an RIL population derived from DOR364 × G19833, supporting a polygenic mode of inheritance. Njobvu et al. (2020) later identified eight QTLs on chromosomes Pv02, Pv04, Pv06, Pv07, Pv09, and Pv10 in another RIL population (Solwezi × AO-1012-29-3-3A), each accounting for 7.6-14.7% of phenotypic variation. Together, these studies establish that Al tolerance in common bean is controlled by multiple moderate-effect loci and provide a genetic foundation for breeding (López-Marín et al., 2009; Njobvu et al., 2020).

In forage legumes such as Medicago, early QTL studies used restriction fragment length polymorphism markers to identify loci associated with Al tolerance in diploid M. sativa subsp. coerulea, with the long-term aim of introgressing favorable alleles into cultivated tetraploid alfalfa (Sledge et al., 2002). Narasimhamoorthy et al. (2005) mapped a major QTL on chromosome 1 that explained 30% of the variation using simple sequence repeat markers. A later study identified three QTLs on linkage groups I, II, and III, accounting for 38%, 16%, and 27% of the variation, respectively (Narasimhamoorthy et al., 2007). In tetraploid alfalfa, Khu et al. (2013) detected three QTLs on linkage groups 1, 4, and 7, explaining 20.8%, 15.2%, and 21.7% of the variation, respectively. These studies collectively indicate that Al tolerance in Medicago is polygenic, with loci distributed across multiple linkage groups.

In *Arabidopsis thaliana*, linkage analysis using an RIL population identified two major QTLs that co-segregated with Al-activated malate efflux, providing an important link between genotype and physiological mechanism (Hoekenga et al., 2003). Subsequent work identified AtALMT1 (At1g08430), which encodes an Al-activated malate transporter and is essential for Al tolerance, although it does not correspond to the major QTL on chromosome 1 (Hoekenga et al., 2006). These findings were instrumental in establishing the mechanistic basis of Al detoxification through organic acid exudation.

Taken together, classical QTL mapping across a wide range of species showed that Al tolerance is generally governed by a combination of major-effect loci and numerous minor genes. These studies also enabled the identification of key gene families, especially ALMT and MATE, that underlie major detoxification mechanisms (Kochian et al., 2015). A visual summary of the major QTLs and causal genes identified through classical linkage mapping is provided in **Figure 2**.

### Limitations of biparental QTL mapping

Although biparental QTL mapping has made foundational contributions, it is constrained by several inherent limitations. Because only two parental lines are involved, the scope of detectable allelic variation is narrow and excludes potentially important alleles present in broader germplasm. Mapping resolution is also limited by the relatively small number of recombination events, with QTL intervals often spanning 10–30 cM and encompassing many candidate genes. In addition, the statistical power of typical mapping populations is often insufficient to detect minor-effect loci or epistatic interactions reliably (Korte and Farlow, 2013). These limitations prompted the adoption of complementary approaches such as GWAS and meta-QTL analysis, which can exploit greater allelic diversity and provide finer resolution. A comparative summary of the principal genetic approaches, including QTL mapping, GWAS, CRISPR/Cas9 editing, MAS, and GS, together with their respective strengths and limitations, is provided in **Table 2**.

**Table 2:** Comparison of genetic dissection methods for aluminum tolerance in major crops.

Method	Suitable population	Advantages	Limitations	Representative crop applications
Classical linkage analysis/QTL mapping	Biparental segregating populations (F_2_, RILs, DH)	No reference genome required; detects major-effect QTLs; enables genetic map construction	Low resolution (10–30 cM); only captures allelic differences between two parents; limited power for minor loci	Wheat (4DL), maize (2L), barley (4H)
Genome-wide association study (GWAS)	Natural diversity panels (hundreds to thousands of accessions)	High resolution (near single-nucleotide); detects multiple alleles; can identify rare variants	False positives due to population structure; requires large sample sizes; phenotyping reproducibility critical	Rice (48 loci), barley (Tibetan wild), maize (CNV)
CRISPR/Cas9 genome editing	Any transformable genotype	Precise modification of endogenous genes; can create transgene-free lines; enables fine-tuning of expression	Off-target effects; requires efficient transformation; possible growth-tolerance trade-offs	*Arabidopsis (RAE1/RAH1), soybean (base editing), rice (promoter editing potential)*
Marker-assisted selection (MAS)	Breeding populations	Highly efficient for major genes; low cost; co-dominant markers available	Ineffective for polygenic traits with many small-effect loci	Wheat (TaALMT1 promoter markers), maize (ZmMATE1 CAPS)
Genomic selection (GS)	Training population + breeding population	Captures all small-effect loci; suitable for complex polygenic traits	Requires high-density markers and statistical models; prediction accuracy depends on training set	Rice (upland), Arabidopsis (explains ~70% of variance)

## Genome-wide association studies: capturing natural allelic variation

Genome-wide association studies take advantage of historical recombination accumulated in natural populations to associate genetic polymorphisms with phenotypic traits at high resolution, often approaching the level of single genes or even individual nucleotides. By using diverse germplasm panels that typically contain hundreds or thousands of accessions, GWAS has become a powerful method for dissecting the genetic basis of Al tolerance in crops and model plants alike (Korte and Farlow, 2013; Channapur et al., 2026a).

### GWAS applications in major crops

Rice has been a leading model for GWAS of Al tolerance. Using 383 diverse accessions, Famoso et al. (2011) revealed strong subpopulation differentiation, with japonica accessions showing approximately twice the Al tolerance of indica and aus groups. Their GWAS identified 48 genomic regions, most of which were specific to individual subpopulations. Significant associations were detected at STAR2 across the full population using principal component analysis, and at Nrat1 within the aus subgroup, where haplotype analysis explained roughly 40% of the phenotypic variation and nonsynonymous mutations were predictive of sensitivity. In parallel, biparental QTL mapping identified a major locus encompassing ART1 on chromosome 12, underscoring the complementarity of GWAS and linkage analysis. Subsequent higher-resolution GWAS studies expanded the list of candidate genes. Zhang et al. (2019a), using 3.8 million SNPs and Ting’s core collection of 150 landraces, identified 17 known Al tolerance genes, including Nrat1, ART1, and STAR1, together with 69 novel candidates. Among these, 20 showed differential expression under Al stress, and coding sequence variation was confirmed between tolerant and sensitive varieties. Zhao et al. (2018), using SLAF-seq to generate 67,511 SNPs, detected 25 QTLs explaining 7.27-13.31% of the variation, including loci overlapping STAR1 and OsFRDL4. Overall, these studies demonstrate how GWAS, especially when integrated with transcriptomic and QTL data, can illuminate the complex genetic architecture of Al tolerance in rice.

In barley, GWAS has identified both conserved loci and variation specific to particular germplasm groups. Cai et al. (2013) reported two loci unique to Tibetan wild barley, bpb-9458 on 2H and bpb-8524 on 7H, which explained 12.9% and 9.7% of the variation, respectively, along with a shared locus, bpb-6949 on 4H, located only 0.8 cM from HvMATE, a known determinant of citrate efflux. Linkage disequilibrium decayed more rapidly in wild barley than in cultivated barley, suggesting greater mapping resolution in wild populations. Zhou et al. (2016), working with 218 barley accessions evaluated in two acid-soil environments, identified 22 QTLs through GLM and MLM analyses. Of these, QTL1 on 1H and QTL5 on 4H were consistently detected across environments, and the latter was marked by bPb-8013, the closest marker to HvAACT1. The observed genome-wide linkage disequilibrium decay distance was 3.13 cM (r2 = 0.1), indicating sufficient marker coverage for association analysis.

Maize studies have provided particularly important insight into structural and sequence variation associated with Al tolerance. Maron et al. (2013) demonstrated that copy number variation at the ZmMATE1 locus underlies a major QTL on chromosome 6. Three tandem copies of ZmMATE1, present only in a few inbred lines originating from acidic soils in South America, resulted in elevated gene expression and superior tolerance, suggesting relatively recent adaptive selection. In a segregating F_2_ population, individuals simultaneously carrying elite alleles of ZmASL and SAHH showed an approximately 30% increase in net root growth compared to those without, suggesting that combining multiple small-effect loci could be beneficial (Krill et al., 2010). However, this observation came from natural recombination in a biparental population rather than from empirically constructed pyramided lines, and thus requires further validation through targeted gene pyramiding.

In wheat, the complexity of the hexaploid genome presents additional challenges, but GWAS has still successfully identified loci linked to Al tolerance. Raman et al. (2010) analyzed 1,055 accessions using 178 Diversity Arrays Technology markers and detected associations on 15 chromosomes, confirming known regions such as 4D and 4B while also revealing new loci. Markers targeting the TaALMT1 promoter explained most of the phenotypic variation. Pereira et al. (2015), working with 300 Brazilian wheat genotypes, found that TaALMT1 promoter Types V and VI, present at frequencies of 71.3% and 11.9%, respectively, were associated with greater Al resistance, whereas a Sukkula-like transposon insertion in the TaMATE1B promoter, although present in 80 genotypes, did not consistently improve root growth. More recently, Zeng et al. (2023) combined GWAS with transcriptome profiling and identified a novel QTL on chromosome 1A, represented by four SNPs explaining 27.2% of the variation, together with 11 candidate genes including E3 ubiquitin ligases such as ATL39-like and ATL4-like. Using 9K and 90K SNP arrays in two Pacific Northwest winter wheat populations totaling about 860 accessions, Froese and Carter (2016) identified 55 loci associated with acidic soil tolerance, 15 of which were shared between the two populations. Notably, the favorable allele of the ALMT1-linked marker wmc331 was rare, suggesting the existence of tolerance mechanisms beyond ALMT1.

In soybean, both classical mapping and GWAS have shown that Al tolerance is genetically complex, with substantial additive and epistatic effects and a large contribution from unmapped minor loci, which together account for 43.07% of the phenotypic variation (Korir et al., 2013). Later GWAS analyses refined this picture by identifying specific candidate genes. Xiang et al. (2022) highlighted Glyma.02g211800, encoding an F-box/leucine-rich repeat protein, and Glyma.20g185500, encoding a bHLH transcription factor, as promising candidates. Wang et al. (2019), integrating QTL mapping with transcriptomics, identified Glyma.04g218700, a WRKY transcription factor strongly induced by Al stress, for further functional validation. Functional studies have also uncovered new regulatory mechanisms, including the role of GmSTOP1–3 in enhancing Al tolerance by promoting flavonoid biosynthesis, such as genistein production, thereby reducing reactive oxygen species accumulation (Liu et al., 2024).

In rapeseed, GWAS has identified multiple loci associated with root growth and biomass under Al stress. Gao et al. (2021) detected 13 SNPs linked to relative root length and relative dry weight across ten chromosomes, with candidate genes including MATE transporters and hormone-related regulators such as JAZ and GA2OX. Zhou et al. (2022), integrating GWAS with transcriptome analysis, identified 43 significant SNPs and 777 candidate genes, of which 64 were differentially expressed under Al stress, including transporters and genes related to cell wall modification. These studies, together with the report by Du et al. (2022) of five QTLs each explaining 5.96-7.02% of the variation, support a polygenic basis for Al tolerance in rapeseed. It is also noteworthy that GWAS in Brassica napus has been extended to other metal toxicities, such as manganese, leading to the identification of BnMTP8.A09 as a key tolerance gene (Raman et al., 2025).

Legume GWAS has further broadened our understanding of Al tolerance. In common bean, Ambachew and Blair (2021) detected 15 significant SNP associations for root traits under Al stress, including average diameter, fork number, root volume, surface area, and total length. These associations mapped to chromosomes Pv01, Pv04, Pv05, Pv06, and Pv11, with candidate intervals containing ALMT and MATE transporter genes. In chickpea, Jia et al. (2025) reported the first GWAS for Al tolerance, identifying eight QTLs on six chromosomes, with CaMATE2, CaMATE4, and CaALMT1 emerging as candidate genes. Among these, CaAlt7–2 was associated with both root length under Al stress and the root tolerance index. In Medicago, genome-wide analysis of the ALMT family identified 68 MsALMTs and 18 MtALMTs. MtALMT8 and MtALMT9 were significantly induced by Al in root tips and partially complemented the Al-sensitive phenotype of the Atalmt1 mutant, indicating their functional relevance to Al tolerance (Jin et al., 2024).

In Arabidopsis thaliana, GWAS has enabled particularly high-resolution dissection of regulatory networks involved in acid soil tolerance. Nakano et al. (2020a) identified 140 and 160 SNPs explaining about 70% of the phenotypic variation in Al and proton tolerance, respectively, and uncovered novel genes such as AtTRM28 and AtTRX1. Interestingly, the genetic architectures of Al and proton tolerance were largely distinct. A separate population-specific GWAS in Central Asian accessions showed that a retrotransposon insertion in the AtMATE promoter disrupts STOP1-binding sites, leading to markedly reduced AtMATE expression and lower Al tolerance (Nakano et al., 2020b). Expression GWAS of PGIP1 further identified both STOP1-dependent and STOP1-independent regulatory pathways under Al stress, including roles for a thioredoxin superfamily protein, NAC027, and an R-R-type MYB transcription factor acting through nitric oxide signaling (Agrahari et al., 2021). Collectively, these studies illustrate the utility of GWAS and eGWAS in dissecting the complex signaling networks underlying Al responses.

### Advantages and challenges of GWAS

Relative to biparental QTL mapping, GWAS offers much higher mapping resolution because it exploits historical recombination, often narrowing associated intervals to individual SNPs or small genomic regions. It also allows multiple alleles to be examined simultaneously across diverse germplasm, making it especially useful for identifying favorable variants absent from biparental populations. This approach is particularly effective for detecting regulatory variation, including promoter polymorphisms, intronic insertions, and copy number variation, all of which can influence gene expression and phenotype directly (Famoso et al., 2011; Maron et al., 2013; Cai et al., 2013).

Technical advances and remaining challenges. Despite its power, GWAS faces several technical challenges. First, population structure and relatedness can generate false-positive associations unless they are appropriately accounted for. Mixed linear models (MLM) that incorporate both fixed effects (SNPs) and random effects (kinship matrix) have become standard, but newer methods such as FarmCPU (fixed and random model circulating probability unification) and BLINK (Bayesian information criterion linkage-dis-equilibrium block iterative nested key) offer improved computational efficiency and power (Ahmed et al., 2024). Second, rare alleles (minor allele frequency < 5%) remain difficult to detect without very large sample sizes (typically > 1000 accessions), and imputation using reference panels can help but may introduce errors. Third, phenotyping continues to be a major limiting factor, especially for root-based traits under Al stress, which are labor-intensive to measure and highly sensitive to environmental conditions (Famoso et al., 2010). Inconsistencies in trait definition and measurement can also lead to poor reproducibility across studies and environments, emphasizing the need for standardized phenotyping protocols and robust validation across multiple conditions (Cai et al., 2013; Zhou et al., 2016).

Recent developments. Multi-trait GWAS and multi-environment GWAS (ME-GWAS) are increasingly being applied to dissect the genetic basis of Al tolerance under fluctuating field conditions. Integration of GWAS with transcriptomics (e.g., expression GWAS, or eGWAS) has identified regulatory networks underlying Al responses, as demonstrated in *Arabidopsis* by Agrahari et al. (2021) and in wheat by Zeng et al. (2023). Furthermore, post-GWAS strategies including fine-mapping, haplotype analysis, and CRISPR-based validation have become essential for moving from statistical associations to functional causality (Ahmed et al., 2024). In mung bean, for example, GWAS combined with gene-based association analysis has successfully identified candidate genes for abiotic stress tolerance (Ahmed et al., 2024), providing a model applicable to Al tolerance research. The integration of QTL and GWAS findings across species is also summarized in **Figure 2**.

### CRISPR/CAS9-mediated precise editing: from gene introduction to endogenous fine-tuning

**Figure 3:**
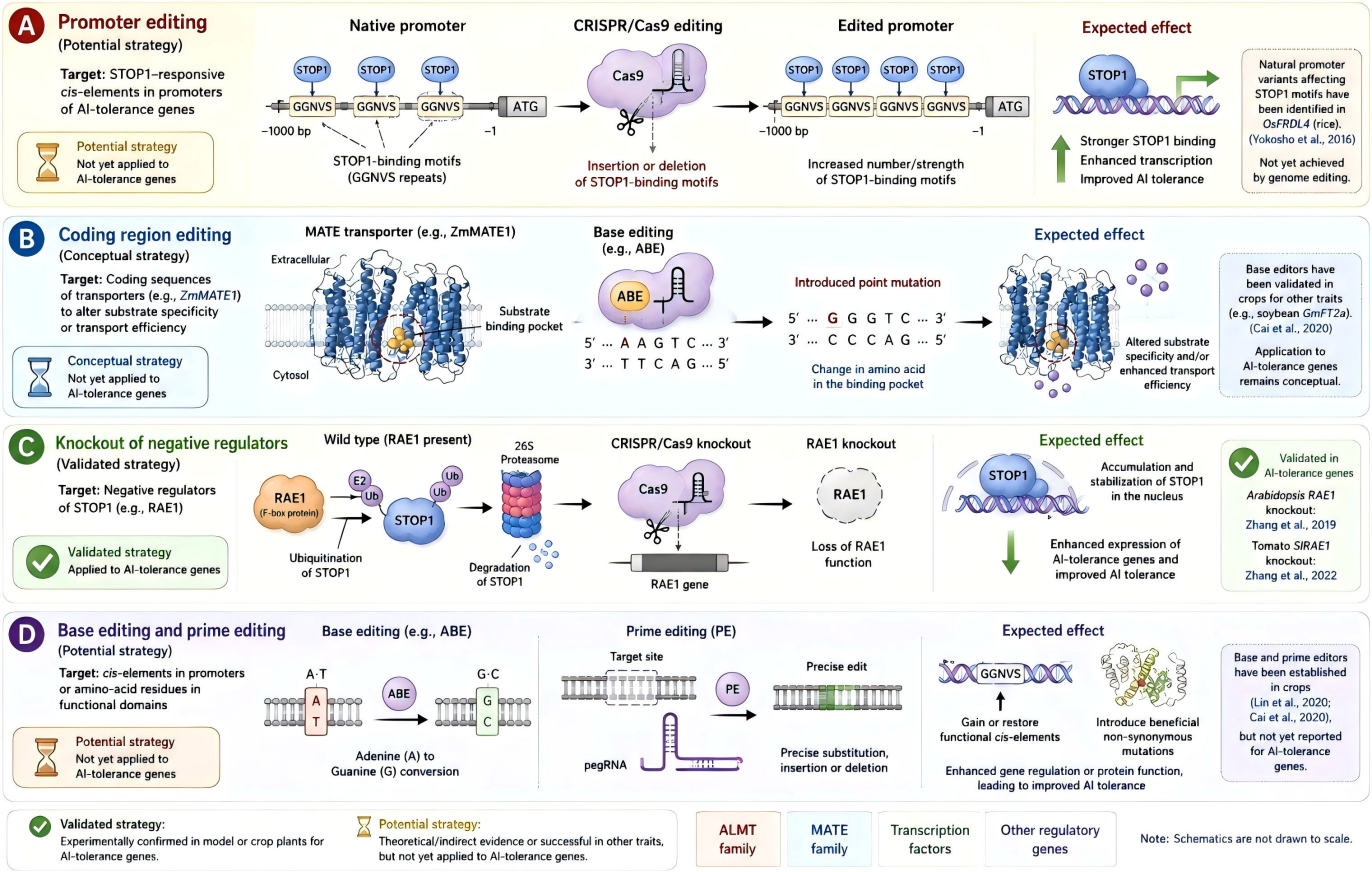
Precision gene-editing strategies (CRISPR/Cas9) for enhancing aluminum tolerance in plants. **(A)** Promoter editing of STOP1-responsive *cis*-elements (potential strategy). CRISPR/Cas9-mediated editing of promoter regions is proposed to enhance transcriptional activation of Al-tolerance genes. STOP1-binding motifs (GGNVS repeats) within promoters can be inserted, duplicated, or modified to strengthen STOP1 binding and increase downstream gene expression. Natural promoter variation affecting STOP1-mediated regulation has been identified in the rice *OsFRDL4* locus, where tandem repeat polymorphisms enhance citrate secretion and Al tolerance (Yokosho et al., 2016). However, direct engineering of such promoter elements for Al-tolerance genes has not yet been reported. **(B)** Coding-region editing of transporter proteins (conceptual strategy). Base editing or targeted mutagenesis of coding sequences may alter transporter activity, substrate specificity, or transport efficiency of Al-tolerance proteins such as MATE transporters (e.g., *ZmMATE1*). Amino acid substitutions introduced within substrate-binding pockets could potentially improve organic acid transport capacity and aluminum detoxification. Similar precision editing approaches have been successfully applied to agronomic traits in crops, including base editing of *GmFT2a* in soybean (Cai et al., 2020), although applications to Al-tolerance transporters remain conceptual. **(C)** Knockout of negative regulators of STOP1 stability (validated strategy). CRISPR/Cas9-mediated disruption of negative regulatory genes such as *RAE1* prevents ubiquitination and proteasomal degradation of the transcription factor STOP1. In wild-type plants, the F-box protein RAE1 promotes STOP1 ubiquitination and degradation via the 26S proteasome. Loss of RAE1 function results in nuclear accumulation and stabilization of STOP1, thereby enhancing transcription of Al-tolerance genes and improving tolerance. This strategy has been experimentally validated in *Arabidopsis thaliana* through *RAE1* knockout mutants (Zhang et al., 2019b) and further extended to tomato via *SlRAE1* editing (Zhang et al., 2022). **(D)** Base editing and prime editing for precision sequence engineering (potential strategy). Advanced genome-editing systems, including adenine base editors (ABE) and prime editors (PE), enable precise nucleotide substitutions, insertions, and deletions without generating double-strand breaks. ABE-mediated conversion of A·T to G·C base pairs or PE-directed sequence replacement could be used to create or restore functional cis-elements in promoters or introduce beneficial missense mutations in transporter functional domains. These technologies have been established in multiple crop species (Lin et al., 2020; Cai et al., 2020), but applications targeting Al-tolerance genes have not yet been reported.

Traditional transgenic approaches, including overexpression of genes such as TaALMT1 and ZmMATE1, have clearly demonstrated that Al tolerance can be improved in crops through genetic engineering. However, their practical deployment has often been limited by regulatory constraints and public acceptance. CRISPR/Cas-based genome editing provides a fundamentally different approach, allowing precise modification of endogenous genes without necessarily introducing foreign DNA. Strategies such as ribonucleoprotein delivery, viral vectors, and transgene-killer CRISPR systems can produce transgene-free edited plants and may therefore ease regulatory barriers (Bhattacharjee et al., 2023). Importantly, genome editing also makes it possible to fine-tune gene expression. In particular, promoter editing offers a way to modify cis-regulatory elements and thereby alter the timing, location, and intensity of endogenous gene expression, rather than relying on constitutive overexpression as in conventional transgenic systems (Tang and Zhang, 2023). The four main CRISPR‑based editing strategies—promoter editing, coding‑region editing, repressor knockout, and base/prime editing—are illustrated in **Figure 3**.

#### Established strategies successfully applied to Al tolerance genes

In *Arabidopsis thaliana*, knockout of the F-box negative regulators *RAE1* and *RAH1* using CRISPR/Cas9 increases STOP1 stability and enhances Al tolerance, although often at the cost of impaired growth, revealing a trade-off between tolerance and development (Zhang et al., 2019b; Fang et al., 2021). These examples represent direct applications of CRISPR editing to Al tolerance genes.

#### Promising strategies with demonstrated potential in crops

Promoter editing is conceptually attractive because natural variation in *cis*-regulatory regions is a major determinant of Al tolerance. In rice, a retrotransposon insertion in the *OsFRDL4* promoter introduces ART1-binding sites, enhancing citrate secretion (Yokosho et al., 2016). Although CRISPR-based promoter editing has not yet been reported for *OsFRDL4*, it has been successfully applied to other genes in rice (Tang and Zhang, 2023). Thus, promoter editing remains a validated strategy awaiting application to Al tolerance.

Base editing allows targeted single-nucleotide substitutions without double-strand breaks. In soybean, a cytosine base editor has been used to generate heritable substitutions in *GmFT2a* and *GmFT4* (Cai et al., 2020). While these are flowering time genes, the platform is directly transferable to Al tolerance genes such as *GmSTOP1-3*. Similarly, prime editing has been demonstrated in rice and wheat for genes like *OsPDS* (Lin et al., 2020), establishing a powerful platform that could, in principle, be applied to recreate natural favorable alleles of *OsFRDL4* or *GmSTOP1-3*.

The current status of each CRISPR-based strategy, whether directly validated in Al tolerance genes, demonstrated for other traits in the same crop, or still conceptual, is summarized in **Table 3**.

**Table 3:** Status of CRISPR/Cas9 editing strategies for improving aluminum tolerance.

Strategy	Directly applied to Al tolerance gene?	Applied to other trait in same crop?	Conceptual proposal	Example reference (Al or other)
Knockout of negative regulators (RAE1/RAH1)	Yes (Arabidopsis)	Yes (rice, maize for other traits)	Yes	Zhang et al., 2019b; Fang et al., 2021
Promoter editing	No	Yes (rice, e.g., OsDLT promoter)	Yes (OsFRDL4 promoter)	Tang and Zhang, 2023
Base editing	No	Yes (soybean GmFT2a)	Yes (GmSTOP1-3)	Cai et al., 2020
Prime editing	No	Yes (rice OsPDS)	Yes (any Al gene)	Lin et al., 2020
Transgene-free delivery (RNP, virus)	No	Yes (wheat, maize)	Yes	Gu et al., 2021

#### Transgene-free genome editing: platforms and regulatory landscape

Three main strategies produce transgene-free edited plants: segregation of transgenes, transient expression of CRISPR components, and DNA-free delivery of ribonucleoproteins (Gu et al., 2021). These methods may simplify regulatory approval, but the regulatory landscape is highly variable across jurisdictions.

The regulatory status of transgene-free edited crops is jurisdiction-dependent (**Table 4**). In the USA, the USDA SECURE rule exempts plants with small deletions or substitutions if no transgene is retained. In the EU, Directive 2023/1762 classifies most targeted mutagenesis products as GMOs, although transgene-free edits may be subject to less stringent requirements if no foreign DNA is present. Major producing countries such as Brazil, China, India, and Japan have established or are developing their own frameworks, ranging from case-by-case assessment to full exemption for SDN-1 edits.

**Table 4:** Regulatory frameworks for transgene−free genome−edited crops in selected jurisdictions.

Country/region	Regulatory body	Key rule/guidance	Classification of transgene-free SDN-1 edits
USA	USDA-APHIS	SECURE rule	Generally exempt if no transgene present
European Union	EFSA	Directive 2023/1762	Regulated as GMO, but simplified procedure possible
Brazil	CTNBio	Normative Resolution No. 16	Exempt if no transgene and no foreign DNA
China	MoA	2022 guidelines	Case-by-case assessment; most SDN-1 not regulated as GMO
India	GEAC	2022 SOP	Exempt if no foreign DNA and no off-targets
Japan	MEXT/MAFF	2019 guidelines	Not regulated as GMO if no transgene

## High-throughput phenotyping

High-throughput phenotyping has become essential in Al tolerance research because accurate, scalable, and reproducible phenotyping remains one of the major constraints on progress. Traditional methods, including manual root length measurement, hematoxylin staining, and calculation of relative root growth, are laborious, low-throughput, and susceptible to operator bias and environmental variation (Clark et al., 2013; Ma et al., 2016). These shortcomings limit the effectiveness of both QTL mapping and GWAS, which depend on precise and consistent phenotypic data (Famoso et al., 2010). To overcome these issues, several high-throughput platforms have been developed. These include automated imaging systems that use cross-polarized light in combination with RootReader2D software, enabling non-destructive and batch-based quantification of whole root systems for large-scale genetic studies (Famoso et al., 2010; Clark et al., 2013). More recently, the HTPRootSlides platform has incorporated an S-shaped circulation mechanism for 141 root boxes, making it possible to capture images at sub-hourly intervals over periods of at least 14 days and thereby greatly improving dynamic root phenotyping (Deng et al., 2025).

### Advances in controlled-environment phenotyping

Automated phenotyping platforms have substantially increased both the throughput and precision of Al tolerance studies. Famoso et al. (2010) developed a high-throughput system that combined a modified hydroponic setup based on Magnavaca’s solution with digital imaging, allowing assessment of entire root systems rather than only the longest root and improving experimental reproducibility.

Clark et al. (2013) further advanced this area by designing a two-dimensional root phenotyping platform that combined high-resolution cross-polarized imaging with the freely available RootReader2D software. This system includes both user-guided and automated tools for extracting multiple root architectural traits, such as total root length, branching pattern, and growth angle, across a wide range of species including rice, maize, sorghum, tomato, and Arabidopsis. Such platforms have enabled large-scale genetic analyses, including GWAS of root growth and Al tolerance, by making complex root phenotypes more accessible to quantitative analysis.

More recently, Channapur et al. (2026b) developed an optimized high-throughput pipeline specifically for profiling root system architecture under Al stress in maize seedlings. Using a standardized hydroponic protocol with 300 μM AlCl_3_ and imaging at 11 days after germination, together with WinRHIZO-based trait extraction, they measured total root length, root surface area, root volume, average diameter, and root tip number in 250 tropical inbred lines. They also derived relative root tolerance indices and percentage reduction metrics to distinguish tolerant from susceptible genotypes. This work illustrates the continuing progression of controlled-environment phenotyping toward greater scalability, precision, and multivariate trait integration.

### Chemical and fluorescent assays for Al detection

Histochemical staining methods have also been adapted for higher-throughput screening. Hematoxylin staining, which yields a blue-purple coloration when bound to Al in root tissues, remains a widely used qualitative assay for evaluating tolerance, as shown in rapid hydroponic screening protocols for barley (Ma et al., 1997) and in tropical maize breeding programs, where it has served as a reliable phenotypic index (Cançado et al., 1999). Fluorescent probes such as morin provide more sensitive detection of Al accumulation (Silva et al., 2013). When coupled with imaging systems or plate readers, both methods can be adapted for semi-quantitative or quantitative analysis, thereby improving objectivity and throughput. These assays offer rapid indicators of Al uptake and localization and complement growth-based phenotyping.

### Field-based and remote sensing approaches

Although controlled-environment assays allow precise measurements, field phenotyping remains indispensable for capturing genotype-by-environment interactions. Recent developments in remote sensing, especially the use of unmanned aerial vehicles equipped with multispectral or hyperspectral sensors, now permit non-destructive monitoring of crop responses to Al stress at large spatial scales. Vegetation indices such as NDVI can detect changes in canopy vigor and chlorophyll content associated with stress.

Hernandez et al. (2022) demonstrated the use of C-band synthetic aperture radar to map Al stress in wheat. They reported a strong negative correlation between SAR backscatter coefficients (σ°VV) and plant height under Al stress (r = −0.84), and they developed a third-order polynomial model to estimate NDVI from σ°VV with an R2 of 0.70. This approach enables spatial mapping of Al stress responses during key developmental stages and provides a valuable means of large-scale phenotyping in the field.

### Integration with advanced genetic populations

Combining high-throughput phenotyping with advanced genetic populations can further enhance trait dissection. Multi-parent advanced generation inter-cross populations, for example, offer greater recombination and allelic diversity than biparental populations, thereby improving mapping resolution. Although such populations have been used mainly for other abiotic stresses, including salinity and sodicity, they have already proven effective for identifying loci associated with stress adaptation (Krishnamurthy et al., 2022). Interestingly, the identification of an ALMT-encoding gene, LOC_Os06g15779, in sodicity tolerance GWAS suggests possible mechanistic overlap between Al toxicity and other soil-related stresses.

### Emerging directions

Future phenotyping strategies are likely to incorporate multi-scale and multi-omics approaches. Single-cell transcriptomics, for instance, has the potential to resolve Al-responsive gene expression at the cellular level and thereby identify specific cell types that play central roles in tolerance. Recent single-cell RNA sequencing studies in Arabidopsis roots have already demonstrated the feasibility of capturing cell-type-specific transcriptional dynamics (Jean-Baptiste et al., 2019). Spatial transcriptomics and *in situ* sequencing add another important dimension by revealing where within tissues those transcriptional changes occur, which is particularly relevant to understanding root responses to Al stress (Moskal et al., 2025). A recent study combining single-cell and spatial transcriptomics in rice roots under soil stress conditions further illustrates the potential of these approaches for uncovering cell-type-specific adaptation mechanisms (Zhu et al., 2025). Together, these technologies are poised to provide unprecedented insight into the cellular basis of Al tolerance (Nobori, 2025).

Overall, high-throughput phenotyping, from automated root imaging to remote sensing and single-cell analysis, is reshaping the study of Al tolerance. Nevertheless, major challenges remain, including the standardization of phenotyping protocols, the integration of data across scales, and the linking of phenotypes to underlying genetic variation. Continued development of robust and scalable phenotyping systems will be essential for fully exploiting genomic tools in crop improvement for acid-soil environments.

## Marker-assisted selection and genomic selection

Even in the absence of genome editing, molecular markers based on natural allelic variation have already been used effectively in breeding programs to improve Al tolerance. Marker-assisted selection has been particularly successful when the trait is governed by major-effect loci, whereas genomic selection offers an alternative for more complex traits controlled by many loci of small effect.

### Successful MAS examples

In wheat, MAS has been widely used on the basis of functional polymorphisms in major Al tolerance genes. PCR-based markers targeting tandem repeat variation in the TaALMT1 promoter are now routinely applied in breeding programs. Pereira et al. (2015) analyzed 300 Brazilian wheat genotypes and identified seven TaALMT1 promoter alleles, designated Types I-VII, among which Types V and VI were strongly associated with enhanced Al tolerance. A transposon insertion in the TaMATE1B promoter was also detected in a subset of genotypes and linked to constitutive citrate efflux (Tovkach et al., 2013). Genotypes combining favorable TaALMT1 alleles with the TaMATE1B insertion showed the greatest root growth under acidic soil conditions (Pereira et al., 2015). Supporting these findings, Froese and Carter (2016) identified SNP markers associated with acidic soil and Al tolerance, facilitating rapid marker-based selection.

In maize, functional markers based on structural variation in ZmMATE1 have been successfully implemented. Cleaved amplified polymorphic sequence markers that distinguish the presence or absence of an intronic retrotransposon insertion allow efficient screening of breeding materials. The favorable allele is associated with greater gene expression and improved performance under acid-soil conditions. Field validation showed that lines and hybrids lacking the favorable ZmMATE1 allele exhibited yield reductions of 18.7% and 14.7%, respectively, under acidic conditions relative to optimal environments (Vasconcellos et al., 2021).

In rice, OsFRDL4 and Nrat1 are well-established determinants of Al tolerance. A promoter variation, such as a transposon insertion, in OsFRDL4 enhances its expression and is associated with the high Al tolerance observed in temperate japonica rice (Yokosho et al., 2016). Haplotype analysis of Nrat1 likewise revealed a susceptible haplotype within the aus subpopulation that accounts for approximately 40% of the phenotypic variation in Al tolerance (Famoso et al., 2011). Such functional variants represent strong targets for marker development in rice breeding.

In soybean, fine mapping of QTLs has enabled the development of functional markers for MAS. Cai et al. (2019) narrowed qAl-HC2 on chromosome 18 to a 69.95 kb region containing a cluster of class III peroxidase genes and identified GmPrx145 as a strong candidate positively regulating Al tolerance in transgenic soybean hairy roots. Abdel-Haleem et al. (2014) also developed a SimpleProbe assay targeting a SNP within a citrate synthase homolog on chromosome Gm08. This marker can distinguish the tolerant allele derived from PI 416937 and facilitates introgression of the major QTLs qAL_HIAL_08 and qAL_PC_08 into elite cultivars.

Together, these examples illustrate that MAS is highly effective when major genes or tightly linked markers are available. Its usefulness is much more limited, however, when the phenotype is determined by many loci with individually small effects.

### Genomic selection: a strategic tool for polygenic Al tolerance

For complex traits such as Al tolerance in many species, particularly soybean, rapeseed, and common bean, where numerous minor QTLs contribute to the phenotype, marker-asssisted selection (MAS) is often ineffective because each individual locus explains only a small fraction of the variation. In such cases, genomic selection (GS) offers a more powerful and strategic alternative. GS uses genome-wide marker information (typically tens of thousands of SNPs) to predict genomic estimated breeding values (GEBVs) based on training population in which both phenotypes and genotypes are known. The predictive model is then applied to breeding population that are genotyped but not phenotyped, thereby accelerating the breeding cycle (Meuwissen et al., 2001; Crossa et al., 2017).

Strategic importance for crop resilience. GS is particularly valuable for developing future crop varieties with enhanced resilience to environmental stresses, including Al toxicity, because: (i) it captures the cumulative effects of many small-effect loci that are individually undetectable by QTL mapping or GWAS; (ii) it can integrate data from multiple correlated traits (e.g., root growth, grain yield, nutrient uptake) through multi-trait GS models; (iii) it enables rapid recycling of elite germplasm without the need for QTL validation; and (iv) it can be combined with envirotyping to predict genotype-by-environment interactions for acid-soil adaptation.

Examples in Al tolerance research. In *Arabidopsis thaliana*, Nakano et al. (2020a) showed that genomic prediction models (using GBLUP and BayesR) could explain around 70% of the phenotypic variance for both Al and proton tolerance. Notably, accessions whose observed phenotypes differed strongly from model predictions were concentrated in particular geographic regions, suggesting local adaptation and the presence of unique alleles not fully represented in the training set, highlighting both the power and a limitation of GS.

In rice, Bartholomé et al. (2024) assessed GS in a synthetic upland population and found that multi-environment models achieved predictive abilities of 0.67 for flowering time, 0.60 for plant height, and 0.53 for grain yield. They estimated that GS could reduce breeding cycle time for acid-soil adaptation from conventional 8–10 years to 4–5 years in their specific upland rice program. It is important to note that such gains are highly dependent on species generation time, transformation efficiency, and field testing requirements, which vary dramatically across crops and breeding programs.

In sorghum, Bernardino et al. (2021) applied GS in a multiparental random mating population developed for adaptation to tropical acid soils in Brazil. Their results showed that GS, especially when incorporating dominance effects and GWAS-derived fixed effects (a strategy known as “GS + GWAS”), could improve prediction accuracy for grain yield, plant height, and Al tolerance. This approach is particularly relevant for tropical regions where acid soils are widespread and where hybrid breeding is common.

Successful implementation of genomic selection for Al tolerance hinges on several practical considerations. The training population must adequately represent the target breeding germplasm and be sufficiently large, typically several hundred individuals (more than 200–300). Prediction models vary in complexity, from simple ridge regression or GBLUP to more advanced approaches such as BayesR, BayesCπ, and machine learning algorithms (random forest, support vector regression, deep learning). Cost−effectiveness also matters: although genotyping costs continue to fall, GS still requires an initial investment in high−density marker data; once the prediction model is validated, however, low−cost imputation or low−density panels may suffice for routine selection. Importantly, GS is not a substitute for MAS or GWAS but rather a complementary tool. For Al tolerance, a tiered strategy can be envisioned: MAS targets major−effect loci (e.g., *ZmMATE1*, *TaALMT1*), GS captures background polygenic effects, and GWAS discovers novel loci that can later be incorporated into GS models. **Table 5** contrasts MAS and GS for Al tolerance breeding.

**Table 5:** Comparison of marker-assisted selection (MAS) and genomic selection (GS) for aluminum tolerance breeding.

Feature	MAS	GS
Genetic architecture suited for	Major QTLs (single or few loci)	Polygenic traits (many small-effect loci)
Marker requirement	Few (1–10 functional markers)	High-density (hundreds to thousands of SNPs)
Training population needed?	No	Yes
Captures epistatic effects?	No	Partially (via non-additive models)
Prediction accuracy for Al tolerance	High (if major gene present)	Moderate to high (0.5–0.7 for grain yield under Al stress)
Breeding cycle acceleration	Moderate	High (can reduce cycle time by 50%)
Cost per sample	Low (PCR-based)	Moderate to high (genotyping by sequencing or array)
Example in Al tolerance	Wheat (*TaALMT1*), maize (*ZmMATE1*), rice (*OsFRDL4*)	Rice (Bartholomé et al., 2024), sorghum (Bernardino et al., 2021), *Arabidopsis* (Nakano et al., 2020a)

Looking ahead, the convergence of GS with high-throughput phenotyping (to generate large, high-quality training data) and envirotyping (to model G×E interactions) promises to greatly accelerate breeding for acid-soil adaptation. As genomic resources expand across more crop species, GS is likely to become a standard tool in breeding programs targeting Al tolerance and other complex stress-adaptive traits.

## Gene pyramiding and multi-gene strategies

Introducing a single gene often provides only partial Al tolerance because Al toxicity disrupts multiple physiological and cellular processes, including root growth, cell wall properties, and intracellular homeostasis. For this reason, combining complementary mechanisms through gene pyramiding has become an increasingly attractive strategy for achieving stronger and more durable tolerance.

One of the most effective approaches is to combine genes involved in organic acid exudation. In wheat, genotypes carrying favorable alleles of both *TaALMT1* and *TaMATE1B* show stronger Al tolerance than those possessing only one of the two, suggesting additive or synergistic interactions (Tovkach et al., 2013; Pereira et al., 2015). This is a concrete example of successful pyramiding.

In maize, integrative analyses suggested that pyramiding favorable alleles across multiple loci could theoretically increase net root growth by approximately 30% (Krill et al., 2010), although this prediction awaits validation in empirically constructed pyramided lines.

Internal detoxification provides a second layer of defense. In rice, the tonoplast-localized half−size ABC transporter OsALS1 sequesters Al into vacuoles (Huang et al., 2012). Before vacuolar loading, Al^3+^ must enter the symplast; the NRAMP-family transporter Nrat1 facilitates this uptake from the apoplast (Xia et al., 2010; Famoso et al., 2011). Additionally, the STAR1/STAR2 ABC complex modifies cell wall composition by supplying UDP-glucose, reducing Al binding to the cell wall (Huang et al., 2009). Pyramiding an exclusion gene (*OsFRDL4*) with a sequestration gene (*OsALS1*) or a cell wall modification gene (*STAR1*) could provide layered protection, though this has not yet been empirically tested.

Fitness costs remain a concern. Organic acid secretion requires carbon investment, and excessive activation of stress-response pathways may compromise growth. STOP1 stability is tightly controlled through RAE1/RAH1-mediated degradation (Zhang et al., 2019b; Fang et al., 2021). Disruption of this balance may increase tolerance but incur growth penalties. Stress-inducible or tissue-specific expression systems (e.g., *VuMATE1* in rice bean; Liu et al., 2013; 2016) can minimize metabolic costs.

Finally, genetic strategies should be combined with agronomic management (liming, phosphorus fertilization, organic amendments, beneficial microorganisms) for sustainable productivity on acid soils (Sopandie, 2025).

## STOP1 ortholog divergence

While STOP1 in *Arabidopsis* and ART1 in rice serve as master regulators, their downstream target gene sets are not entirely orthologous. For instance, ART1 directly regulates *Nrat1* and *STAR1/STAR2*, for which clear functional orthologs regulated by AtSTOP1 are not evident in *Arabidopsis*. Conversely, AtSTOP1 regulates a broader set of genes involved in various stress responses. This divergence implies that engineering the STOP1 cascade for improved Al tolerance may have different pleiotropic effects and requires a species-specific understanding of the network.

## Conclusion and future perspectives

Over the past two decades, substantial advances have been made in elucidating the genetic and molecular basis of Al tolerance. Classical QTL mapping and GWAS have been central to the identification of major genes such as *TaALMT1*, *ZmMATE1*, *OsFRDL4*, *SbMATE*, and *HvMATE*, as well as the natural variants that underlie their function. Integration of GWAS with transcriptomics has further accelerated candidate gene discovery (Zeng et al., 2023; Jia et al., 2025), and recent work in legumes has extended these insights beyond cereals. As highlighted in **Table 1**, the convergence of QTL mapping, GWAS, and functional validation has established a core set of ALMT and MATE transporters as key determinants of Al tolerance across diverse crop species.

Genome editing, especially through CRISPR/Cas systems, now offers powerful opportunities for precise improvement of Al tolerance. Promoter editing, base editing, prime editing, and transgene-free delivery are all feasible platforms, although many have not yet been directly applied to Al tolerance genes. The frequent trade-off between improved tolerance and plant growth indicates that stress-inducible or tissue-specific regulatory strategies will be needed.

To guide future research, QTL mapping, GWAS, and CRISPR/Cas editing are highly synergistic. QTL mapping or GWAS can first pinpoint causal genes or regulatory variants, after which CRISPR/Cas can be used to recreate or fine-tune those naturally favorable alleles in elite backgrounds. Conversely, editing-based validation of candidate genes can feed back into more accurate GWAS models. Future breeding strategies should integrate all three layers: discovery by QTL/GWAS, validation and refinement by editing, and deployment by MAS or GS.

Progress in phenotyping is reducing one of the major bottlenecks. Automated root imaging, UAV-based remote sensing, and single-cell/spatial transcriptomics are providing unprecedented resolution. However, challenges remain in standardizing protocols, integrating data across scales, and distinguishing Al-specific stress from co-occurring stresses in the field.

Another critical aspect that requires further investigation is the multi-generational phenotypic stability of CRISPR/Cas-edited lines. Most current studies report only the T_1_ or T_2_ generation under controlled conditions. Whether edited tolerance phenotypes remain stable under field conditions across multiple growing seasons, and whether editing-induced changes may lead to unintended pleiotropic effects or epigenetic alterations that become manifest only after several generations, remains to be determined. Future editing studies should include longitudinal phenotyping of T_3_ and T_4_ generations under both controlled and field acid-soil conditions, coupled with whole-genome resequencing and methylome analysis.

Genomic selection and gene pyramiding are likely to become increasingly important. MAS remains highly effective for major loci, wile GS offers a broader framework for polygenic traits. Pyramiding multiple tolerance mechanisms has the potential to generate synergistic improvements, provided that fitness costs are carefully managed.

Finally, genetic improvement alone will not ensure productive use of acid soils. Integrated strategies combining molecular breeding with agronomic practices such as liming, nutrient management, and soil amendment are essential (Sopandie, 2025). As cultivation expands into regions with extensive acid soils, coordinated strategies that integrate genetics, agronomy, and environmental management will be increasingly necessary.

Future research will be driven by single-cell omics, synthetic biology, and advanced genome editing, potentially enabling the development of “smart” crops that activate Al tolerance only under stress conditions.
